# Targeting of protease activator receptor-2 (PAR-2) antagonist FSLLRY-NH2 as an asthma adjuvant therapy

**DOI:** 10.1097/MD.0000000000022351

**Published:** 2020-10-23

**Authors:** Marinel Ocasio-Rivera, Frances Marin-Maldonado, Geraline Trossi-Torres, Angely Ortiz-Rosado, Valerie Rodríguez-Irizarry, Eric Rodriguez-Lopez, Sahayra Martínez, Sharilyn Almodóvar, Edu Suarez-Martínez

**Affiliations:** a University of Puerto Rico-Ponce, Ponce Puerto Rico; b Ponce Health Science University, Ponce Puerto Rico; c Texas Tech University Health Science Center, Lubbock, TX.

**Keywords:** asthma, PAR-2, PAR-2 antagonist FSLLRY-NH2, protease activated receptor 2

## Abstract

Asthma is a chronic inflammatory and multifactorial respiratory tract disease. It affects over 18 million adults and 6 million children in the USA with Puerto Ricans showing the highest prevalence (12%–19%). This airways illness can be triggered by an environmental stimulus such as grass pollen, fungi spores, cockroaches allergens, dust mites metabolic compounds, and importantly, by environmental proteases such as trypsin and tryptase. Because of the pivotal role of proteases in the onset of asthma pathophysiology, we focused this study on the serine Protease Activated Receptor-2 (PAR-2), a G-protein-coupled receptor widely expressed in cells across the respiratory tract. Herein, we measured the activation of PAR-2 on primary pulmonary bronchial/tracheal epithelial cells, human small airway epithelial cells, lung bronchial smooth muscle cells (with and without asthma). We tested human-derived eosinophils from 61 Puerto Rican participants (33 asthmatic and 28 non-asthmatic). As surrogate of PAR-2 activation or inhibition we used intracellular calcium mobilization assay. We hypothesized that following exposure of the PAR-2 agonist (AC264613), the studied human primary cell types will increase the mobilization of intracellular calcium levels. In contrast, we expected a decrease of the intracellular calcium levels upon exposure to a PAR-2 antagonist (FSLLRY-NH2). The Puerto Rican-derived eosinophils were analyzed for the proinflammatory markers MAPK/PI3K using flow cytometry (n = 8). As expected, the PAR-2 agonist significantly increased the activation of PAR-2 on the bronchial/tracheal epithelial cells, bronchial smooth muscle cells and human small airway epithelial cells (*P* = .01). The PAR-2 antagonist significantly decreased the intracellular calcium levels of these lung primary down to undetectable levels (*P* = .01). Remarkably, the asthmatic-derived eosinophils showed a striking 300% increase of intracellular calcium mobilization suggesting a severe response to the PAR-2 agonist stimuli in asthmatics. In contrast, there were no significant changes between groups after adding the PAR-2 antagonist. Our outcomes revealed that PAR-2 antagonist effectively inhibited the studied primary cells, expecting to decrease the immune response of eosinophils. Most importantly, our results reveal a promising role for the PAR-2 antagonist in targeting bronchial/tracheal epithelial cells, human small airway epithelial cells and bronchial smooth muscle cells with the potential to oblige an asthma adjuvant therapy.

## 1 Introduction

Asthma is a complex chronic inflammatory disease of the airways, varying in severity from occasional episodes of wheezing and shortness of breath to an irreversible, life-threatening lung obstructive disease. The CDC Behavioral Risk Factors Survey have consistently indicated that the lifetime prevalence of asthma in Puerto Rico (PR) is the highest in the USA and its territories with a 12% to 19% for the latest cohort.^[1]^ Moreover, the Puerto Rican population shows inconsistent responses to pharmacological treatments compared to other ethnic groups.^[1,2]^ As a multifactorial disease, many underlying causes of asthma remain unclear. Certainly, interactions occurring between genetic profiles and environmental factors contribute to its clinical manifestations. Importantly, the exposure to specific thresholds levels of allergen(s) has been identified for the severity and disparity of asthma in various populations worldwide, including grass pollen, fungi spores, cockroach allergens, and dust mites products. Moreover, enzymatic activity of environmental proteases such as trypsin and tryptase are associated with multiple pro-inflammatory and immunological responses characterized in the pathology of asthma.^[3]^ For example, the Protease Activated Receptor-2 (PAR-2), a serine protease and autocatalytic G-protein-coupled receptor (GPCR) is widely expressed in human cells across the respiratory tract including airway epithelial and smooth muscle cells, bronchial vascular and epithelial cells, type II pneumocytes, terminal bronchial epithelium, eosinophils, neutrophils, and mast cells.^[4–9]^ All these cells play an important role in triggering, development, clinical manifestations, and severity of asthma. Trypsin and mast cell tryptase caused bronchoconstriction in animal models via the PAR-2 receptors.^[10,11]^ Also, allergic mice exposed to trypsin showed increased the expression of PAR-2 leading to eosinophil infiltration, airway hyper-reactivity, and inflammation considering PAR-2 a pro-inflammatory trigger.^[10]^ However, studies of human PAR-2 at the cellular and molecular level are limited. Herein, we evaluated the potential of PAR-2 antagonism as a therapeutic cell specific target or adjuvant treatment for the health disparity of asthma. In fact, our findings provide insights on the contribution and role of PAR-2 agonist hypersensitivity on eosinophils derived from asthmatic participants. At the respiratory tract level, the outcome of the PAR-2 effectiveness, also open new projections for the development of a future delivery method such as an inhaled or aerosolized drug formulation. The PAR-2 antagonist will act reaching the respiratory cells, acting as potential treatment for the inhibition of the receptor signaling pathway by a reduction or blockage of the characteristic asthma-associated symptoms.

## 2 Methods

### 2.1 Cell culture

We seeded Bronchial/Tracheal Epithelial Cells (cat. PCS-300–014, ATCC, Virginia), Human Small Airway Epithelial Cells (HSAEC) (cat. PCS-301-010, ATCC, Virginia) and Bronchial Smooth Muscle Cells (BSMC) (cat. CC-2576, Lonza Clonetics, Switzerland) on T-75 flasks with the appropriate media and incubated under standard conditions (37°C, humidity 100% and 5% CO2) until reaching confluence of 90% to 95%. Then we trypsinized and seeded a total of 20,000 cells per well in triplicates (96-well plate) of each respective cell type and per treatment, with subsequent use of the Fluo-4 Direct Calcium Assay Kit (cat. F10471, Invitrogen, California).

## 3 Participants

### 3.1 Patient confidentiality

This study was reviewed and approved by the Ponce Medical School Foundation Internal Review Board constituted by the following members: Simón E. Carlo, M.D., Chairman, Idhaliz Flores, Ph.D., Vanessa Rivera Amil, Ph.D., Victoria Michelen, M.D., Luisa Morales, Ph.D., Mary Rodríguez, Psy.D., Axel Ramos, PhD, Eliut Rivera, Ph.D., Mrs. Mardie Geiser, Ms. Elena Vélez, and Mrs. Migdalia Cruz. Following the established confidentiality protocols for the privileged participants information. Participant consent was given prior to start the studied tests, including the Health Insurance Portability and Accountability Act (HIPAA) enforcement. As a case-control study, we surveyed 216 candidates (August 2017 - March 2018) who were evaluated under the following criteria: Inclusion- a) All participants (asthmatics and non-asthmatics) must be Puerto Ricans residing in Puerto Rico; b) 3 generations of Puerto Ricans; c) Asthmatic participants must be physician-diagnosed; d) ages from 5 5 to 62 years; *Exclusion*- physician diagnosis for one of these illnesses: a) chronic bronchitis; b) pulmonary emphysema; c) congestive heart failure; d) cough secondary to drugs such as beta-blockers; e) chronic obstructive pulmonary disease (COPD); f) mastocytosis; g) parathyroid disease; h) recent allergic reaction; or i) any other chronic and inflammatory medical condition that restrict their daily activities. In the present study, we enrolled 67 qualifying participants; of these, we were able to isolate eosinophils from 61 subjects (33 asthmatic and 28 non-asthmatics). The male to female ratio was 1:1.77 (36% males and 64% females). The mean age was 30 ± 1.74 years.

### 3.2 Blood sampling and eosinophil purification

The eosinophil purification assay was performed using 20 ml of K2EDTA-anticoagulated blood samples from participants within 24 hours after phlebotomy. We used the Human Eosinophil Enrichment Kit Stem (Cell Technologies #19256, Canada) and followed the manufacturer protocol as described. This system isolates the eosinophils from other granulocytes by negative selection, obtaining approximately one million cells per participant.

### 3.3 Calcium assay

**Figure 1:**
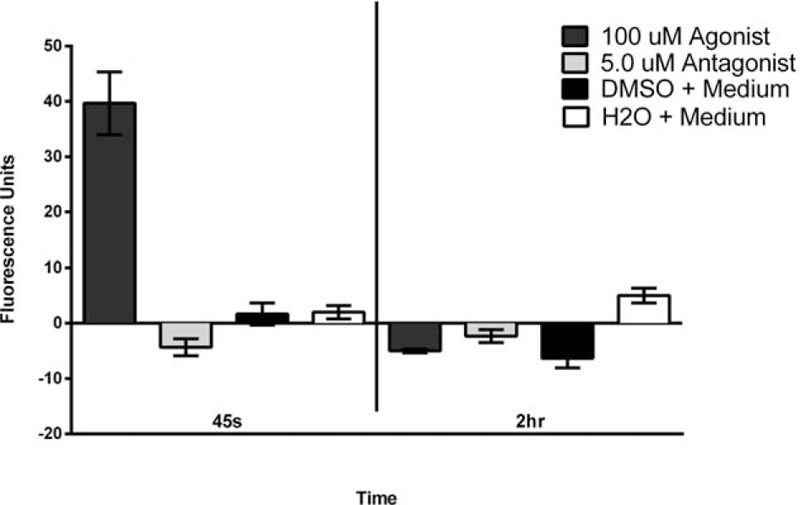
Determination of the time required to detect Intracellular Calcium Mobilization using the Fluo-4 Direct Calcium Assay Kit after adding the PAR-2 agonist to primary bronchial/tracheal epithelial cells. The standard 2-hour incubation recommended when using the intracellular calcium mobilization kit was optimized for our experimental conditions, cell, and receptor types to 45 seconds as the optimal incubation time. Based on the determined time, we titrated for the PAR-2 agonist and PAR-2 antagonist concentrations fitting our experimental conditions resulted at 10 μM for agonist and 0.5 μM for agonist.

We used the Fluo-4 Direct Calcium Assay Kit (Invitrogen #F10472, Massachusetts) to determine the intracellular calcium mobilization as well-established indicator of PAR-2 activation or inhibition of the 4 selected primary cell types. We seeded 20,000 cells/well in 96-well plates. We optimized the last incubation time from 2 hours (suggested) to 45 seconds after exposure to the treatments, PAR-2 agonist AC264613, and PAR-2 antagonist FSLLRY-NH2, due to the rapid kinetics of the PAR-2 activation (Fig. 1.) The intracellular calcium level was measured in triplicates and 3 repetitions by fluorescence at 485/20, 528/20 in relative fluorescence units (RFU), in a BioTek Synergy HT (Wisnooski, Vermont).

### 3.4 Flow cytometry

We used the Muse Cell Analyzer (7200150462, Austin, Texas) and the Muse P13K/ MAPK Dual Pathway Activation Kit (MCH200108, Texas) protocol to determine these markers on the human derived eosinophils was measured in triplicates and 2 repetitions.

### 3.5 Statistical analysis

Data analyses were performed using software SPSS v.26.0 and Graph Pad Prism 6. The significance level was established at *P* < .05. Clinical variables of cases and controls were compared by the X^2^ test with 1 df (degree of freedom); we also used parametric and nonparametric tests as applicable.

## 4 Results

We used an intracellular calcium mobilization assay as indicator of the activation of PAR-2 associated signal transduction. Using the Fluo-4 Direct Calcium Assay Kit we optimized the acquisition parameters to our assay, in terms of incubation times prior to collect the data from the fluorometric readings and the PAR-2 agonist and antagonist concentrations (not shown). Based on our optimization experiments, the ideal experimental time for our cell type for PAR-2 activation and detection of intracellular calcium was 45 seconds (Fig. 1); the PAR-2 agonist and antagonist concentration ideal for our experimental conditions as 10 μM for the agonist and 0.5 μM for antagonist (data not shown).

**Figure 2:**
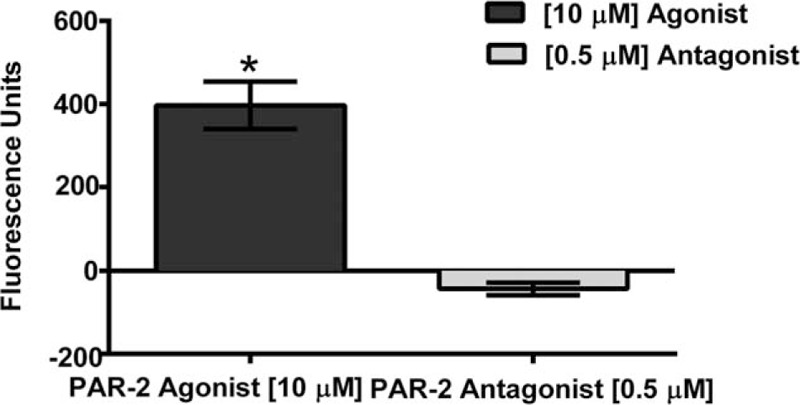
Change in Intracellular Calcium Mobilization in Primary Bronchial/Tracheal Epithelial Cells following treatments with PAR-2 agonist and PAR-2 antagonist. There was a significant decrease up to undetectable levels (*P* = .01) of the intracellular calcium mobilization on Bronchial/Tracheal Epithelial Cells, once exposed to the PAR-2 antagonist FSLLRY-NH2 compared to agonist.

**Figure 3:**
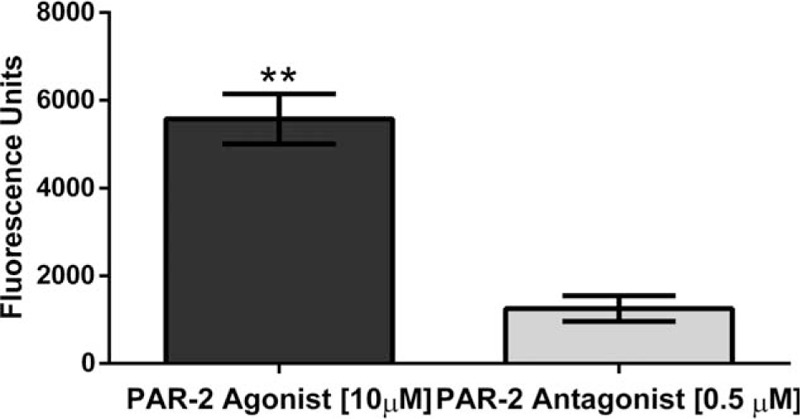
Changes in Intracellular Calcium Mobilization in Human Small Airway Epithelial Cells. There was a significant decrease (*P* = .0024) of intracellular calcium mobilization on Human Small Airway Epithelial Cells, when exposed to the PAR-2 antagonist.

**Figure 4:**
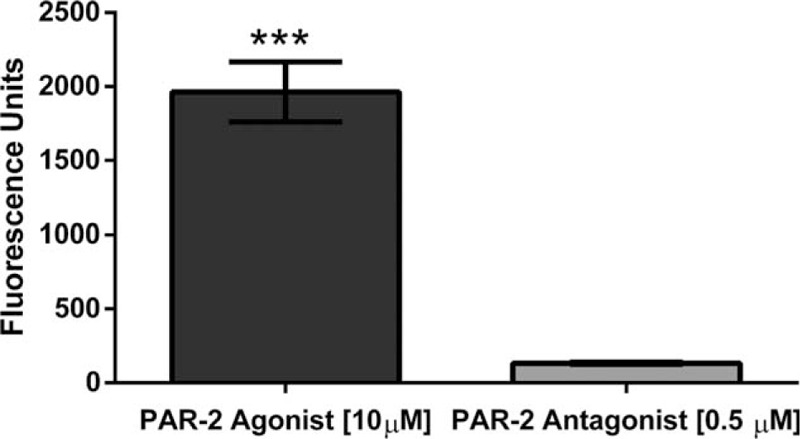
Determination of Intracellular Calcium Mobilization on Primary Bronchial Smooth Cells. There was a significant decrease (*P* = .0001) of intracellular calcium mobilization on Bronchial Smooth muscle cells, when exposed to the PAR-2 antagonist.

**Figure 5:**
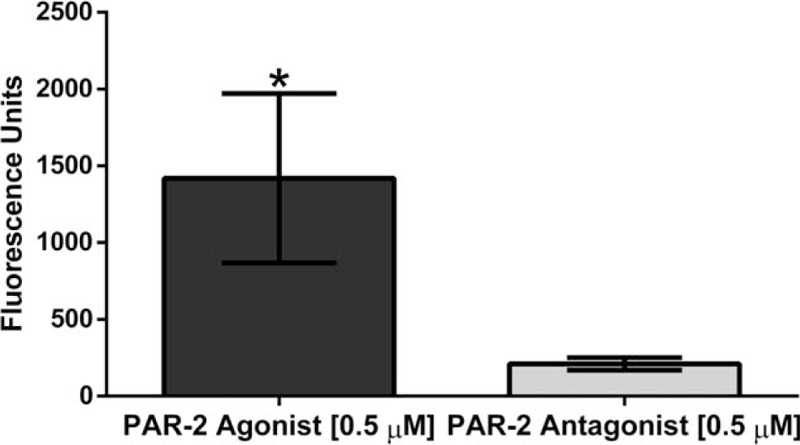
Determination of Intracellular Calcium Mobilization on Primary Bronchial Smooth Cells derived from an asthmatic origin. There was a significant decrease (*P* = .01) of intracellular calcium mobilization on asthmatic-derived Bronchial Smooth muscle cells, when exposed to the PAR-2 antagonist.

**Figure 6:**
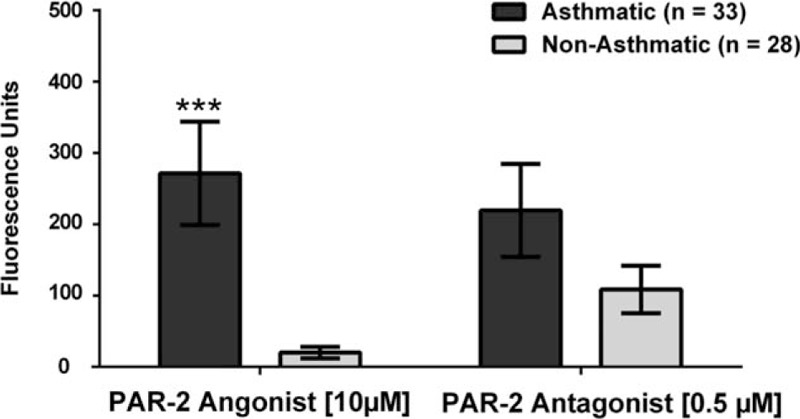
Changes in Intracellular Calcium Mobilization on Human-Derived Eosinophils of Asthmatic and Non-Asthmatic Participants. There was a highly significant difference of (*P* = .0001) of intracellular calcium mobilization on eosinophils between the asthmatic and non-asthmatic participants, when exposed to the PAR-2 agonist. On the contrary, there was no significant difference (*P* = .99) between the case and control groups, when exposed to the PAR-2 antagonist.

Once we established the best parameters, we measured the intracellular calcium mobilization in the presence of each treatment. Our results showed a significant increase in intracellular calcium mobilization in Bronchial/Tracheal Epithelial Cells (*P* = .01)(Fig. 2), Human Small Airway Epithelial Cells (*P* = .0024)(Fig. 3), Bronchial Smooth Muscle Cells (*P* = .0001)(Fig. 4) and Diseased Asthmatic Bronchial Sooth Muscle Cells (*P* = .01)(Fig. 5) when exposed to the PAR-2 agonist. In contrast, PAR-2 expression was inhibited by showing significant decrease when we added the antagonist to these primary cells (Figs. 2–5). Since asthma prevalence in Puerto Ricans is the highest in the Nation, we further explored the intracellular calcium mobilization in eosinophils derived from this population upon exposure to PAR-2 agonist and antagonist treatments by conducting a pilot case-control study with 33 asthmatic and 28 non-asthmatic participants. In the presence of the PAR-2 agonist, the eosinophils derived from the asthmatic group showed a significant 300% increase in PAR-2 activation when compared to the non-asthmatics (*P* < .0001). Treatment with the PAR-2 antagonist, however, did not show significant decrease in the PAR-2 activation. (Fig. 6).

**Figure 7:**
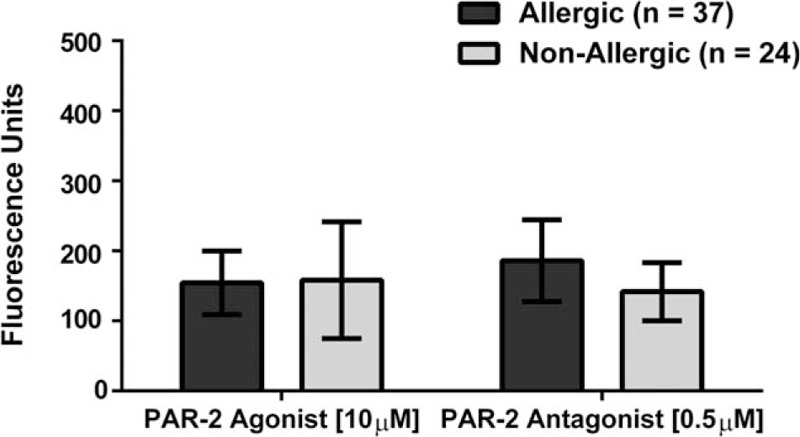
Changes in Intracellular Calcium Mobilization in Human-Derived Eosinophils of Allergic and Non-Allergic Participants. There was no mobilization of intracellular calcium on human-derived eosinophils when comparing allergic and non-allergic participants. Neither treatment showed significant difference (*P* = .10).

**Figure 8:**
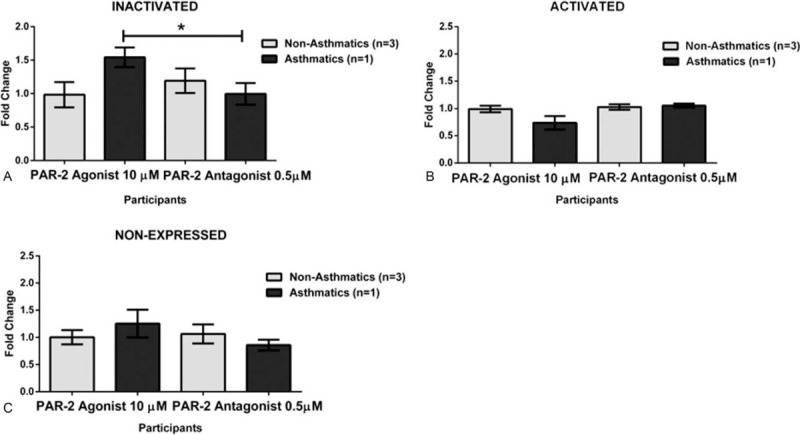
PI3K Changes on Human-Derived Eosinophils from Asthmatic and Non-asthmatic Participants. A. We observed a significant decrease of *P* = .01 when the eosinophils of the asthmatic group were exposed to the PAR-2 antagonist. These cells showed lower inactivation for the PI3K marker. B. There was not a significant difference (*P* = .6465) between the studied groups when the exposed to the PAR-2 antagonist suggesting that the tested cells were expressing (Activated) PI3K marker at the same level. C. There was not a significant difference (*P* = .6897) between the case-control groups upon exposure to the PAR-2 antagonist indicating that at that time PI3K marker was not detected.

**Figure 9:**
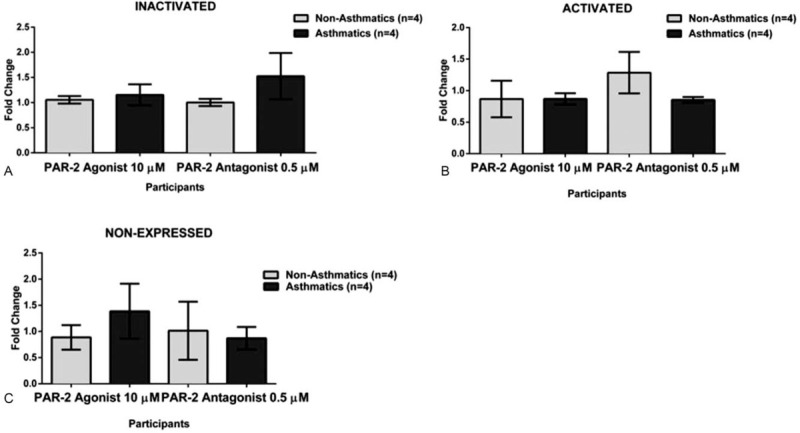
MAPK Changes on Human-Derived Eosinophils from Asthmatic and Non-asthmatic Participants. A. There was not a significant difference (*P* = .1291) between the asthmatic and non-asthmatic groups when the exposed to the PAR-2 antagonist, showing that the cells were not activated (Inactivated) for the MAPK marker at that time point. B. There was not a significant difference (*P* = .5180) between the studied group when exposed to the PAR-2 antagonist indicating that cells were expressing (Activated) MAPK marker at the same level. C. There was not a significant difference (*P* = .7769) between the eosinophils derived from asthmatic and non-asthmatic, when exposed to the PAR-2 antagonist on cells lacking expression (Non-Expressed) of the MAPK marker.

To evaluate whether the observed significant increase of intracellular calcium in eosinophils was due to the asthma, we further stratified the cohort in allergic and non-allergic participants. Our results did not show significant activation (increase in intracellular calcium) nor inhibition (decrease of intracellular calcium) upon exposure to any of the treatments (Fig. 7.). Finally, we tested for the proinflammatory markers mitogen-activated protein kinase (MAPK) and Phosphoinositide 3-kinases (PI3K) to better understand the inflammation processes after PAR-2 is activated, but a limited subset of the biospecimens was available (4 asthmatics and 4 non-asthmatics). Based on our results, none of the PAR-2 treatments changed the expression of MAPK nor PI3K in these cells (Figs. 8 and 9).

## 5 Discussion

The PAR-2 receptor belongs to the seven-transmembrane domain G-Protein Coupled Receptors (GPCR's) including 4 serine protease-activated receptors (PARs): PAR-1–4. This proteolytic receptor is activated by exogenous, environmental, or endogenous serine proteases such as trypsin and tryptase.^[12]^ PAR-2 is cleaved at the extracellular N-terminus domain, triggering a conformational change and directing the tethered ligand to bind to the second extracellular loop domain.,^[12]^ which starts the signal transduction. Although the other members of this family PAR-1, PAR-3 and PAR-4 have been extensively studied, PAR-2 is not entirely deciphered at the immune and inflammatory level. Only PAR-1 is been considered in Food and Drug Administrator (FDA) clinical trials for treatment of platelets-related diseases including cardiovascular events and multiple inflammatory disorders. For instance, the lack of PAR-2 scientific literature -especially in humans,^[13]^ creates a significant gap of knowledge and a limitation for the development of novel drugs and therapeutic agents involving PAR-2 signaling pathways for health-associated dysfunctions or diseases.

It is known that PAR-2 is expressed in human respiratory airway epithelial cells, human bronchial vessels, human airway smooth muscle, type II pneumocytes, terminal bronchial epithelium, macrophages, eosinophils, neutrophils, and mast cells.^[4–9]^ Importantly all these are involved in the pathophysiology of asthma. Activated monocytes tested in vitro expressing PAR-2 produce proinflammatory cytokines,^[14]^ enhance airway reactivity, bronchoconstriction, and eosinophil infiltration in animal models.^[15,16]^ Knight and colleagues reported one of the first PAR-2 case-control studies comparing asthmatics and non-asthmatic participants by studying biopsies with immunohistochemistry at the bronchial level, showing a significant overexpression of PAR-2 in asthmatics.^[17]^ On a separate study, Aubier et al, compared the immune cells profile collected from bronchoalveolar lavage fluid by using flow cytometry and measured the airway smooth muscle enlargement from asthmatic and non-asthmatic participants.^[18]^ Still, a single clinical trial is active using PAR-2 agonist in congenital gastrointestinal disease,^[19]^ but none other clinical trial includes PAR-2 and asthma.

Therefore, herein we evaluated the effect of PAR-2 antagonist to decrease the activation of proinflammatory subsequent signal transduction in several relevant cells involved in the pathophysiology of asthma. We determined the effects of PAR-2 agonist AC264613 and PAR-2 antagonist FSLLRY-NH2 on human primary cells from different microenvironments within the respiratory tract, including bronchial/tracheal epithelial cells, small airway epithelial cells, bronchial smooth muscle cells, and patient-derived eosinophils from asthmatics and non-asthmatics groups.

Our choice of the PAR-2 agonist AC264613 is supported by literature indicating its affinity and specificity for PAR-2 without cross reactivity to PAR-1, which structurally similar to PAR-2.^[20,21]^ The PAR-2 antagonist FSLLRY-NH2 was selected due to its very specific antagonism to PAR-2^[22]^ at the cleavage site not occurring on the other member of its family.^[13]^ The intracellular calcium mobilization levels are indicative for a signal pathway activation of GPCRs including PAR-2.^[22]^ In this case, we implemented a fluorescence-based assay for higher sensitivity at the quantification. Since the PAR-2 cleavage site is located at the extracellular cytoplasmic region, once cleaved it is rapidly autoactivated and the signal transduction is triggered at a time frame from 0 to 180 seconds^[22]^ in our case the optimal time was 45 second for data recording.

All 3 human lung primary cells (the bronchial/ tracheal epithelial cells, human small airway epithelial cells and bronchial smooth muscle cells) showed a significant increase on intracellular calcium levels mobilization representative of the PAR-2 activation. We observed the opposite outcomes when the cells were exposed to the PAR-2 antagonist; the levels of intracellular calcium were significantly lower as expected in a reduced or blocked activation. Guided by our results, we were able to find a commercially available diseased counterpart for the bronchial smooth muscle cells derived from an Asthmatic patient. At the end of the experimentation including the bronchial smooth muscle cells derived from an asthmatic patient, the results were consistent with the previously observed at the other 3 lung primary cells. The PAR-2 antagonist significantly decreases the activation by decreasing the intracellular the calcium mobilization. These findings are very relevant to our population, since Puerto Ricans have the highest prevalence of asthma in the U.S.A. and its territories.^[1,23]^

Taking into consideration that PAR2 is the only known functional PAR expressed by eosinophils,^[24]^ we undertook a case-control study with Puerto Ricans-derived eosinophils from asthmatics and non-asthmatic participants. There have been proposed 2 possible mechanisms resulting from PAR-2 activation and its cell signaling. First, PAR-2 tends to induce MAPK signaling pathway^[25]^ and secondly PI3K, both of which have fundamental roles on inflammatory diseases.^[18,19]^ Importantly, the activation of the PI3K pathway, downstream signaling molecule Protein Kinase B (Akt) promotes eosinophil degranulation.^[26]^ We sought to analyze if indeed PAR-2 activated MAPK and PI3K signaling pathways differently in the presence of an agonist or an antagonist. We performed the MAPK and PI3K by flowcytometry analysis and results were not significant although we observed some trends between groups. However, we determined that the number of available and viable eosinophils (4 asthmatics and 4 non-asthmatics), the required the number of replicas, and the short spotting time for capturing the PAR-2 effects were limited to obtain rigorous and reproducible results. Other methods such as Western blot, immunofluorescent assays or live-cell imaging may be considered to detect these markers under our experimental conditions.

Impressively, the eosinophils collected from the asthmatic group and exposed to the PAR-2 agonist showed an overwhelming 300% increase of intracellular calcium mobilization compared to the non-asthmatic group. This finding may represent PAR-2 hypersensitivity of asthmatic-derived eosinophils, or an up regulation of PAR-2 in asthmatic suggesting a direct association of PAR-2, asthma, and these immune cells.^[27]^ The observed outcomes proposed that these granulocytes in asthmatics are affected differently by PAR-2 stimulus than the non-asthmatic derived. In contrast, there were no significant differences within the groups of eosinophils upon exposure to the antagonist. This observation may be attributed to the treatment concentration. There is also a possibility of a PAR-2 up-regulation upon exposure to the FSLLRY-NH2 antagonist or testing another antagonist in these immunological cells. More importantly, the lack of inhibition may also be a consequence of the large beta-arresting-dependent endosomal Extracellular Signal-Regulator Kinases (ERK1/2) that effectively engage large cytoplasmic stores of PAR-2, prone to rapidly externalized when the stimuli are received.^[28]^

In either situation, since the PAR-2 antagonist FSLLRY-NH2 was effective at the tested cells including the asthmatics bronchial smooth muscle cells, we expect that PAR-2 antagonist block the signal transduction, which is implicated in the recruitment of eosinophils. For instance, at this situation circulatory eosinophils will not be considered as a therapeutic target. Our results indicated the potential of this molecule for fast track studies from translational to clinical settings, even more that PAR-2 antagonist FSLLRY-NH2 can be developed as a nebulizer or aerosolized formulation and used as an adjuvant therapy for treatment and managing of asthma. The effect of PAR-2 identified in this study provides the opportunity to perform further studies with this puzzling GPCR signaling.

## Acknowledgments

Special thanks to: Ponce Health Sciences University Staff, PHSU MD student Joedali Baez and undergrad Students from the University of Puerto Rico at Ponce, Emmanuel León-Colón, Gerardo Arroyo, Solymar Feliciano, Nicole de la Rosa, and Maryangie Martinez for technical assistance. Luisa Morales, Dr.P.H. for revision and determination of the statistical analyses.

## Author contributions

**Conceptualization:** Marinel Ocasio-Rivera, Frances Marin-Maldonado, Angely Ortiz-Rosado, Sharilyn Almodovar, Edu B Suarez-Martínez.

**Data curation:** Marinel Ocasio-Rivera, Frances Marin-Maldonado, Geraline Trossi-Torres, Angely Ortiz-Rosado.

**Formal analysis:** Marinel Ocasio-Rivera, Frances Marin-Maldonado, Geraline Trossi-Torres, Valerie Rodríguez-Irizarry, Edu B Suarez-Martínez.

**Funding acquisition:** Edu B Suarez-Martínez.

**Investigation:** Marinel Ocasio-Rivera, Frances Marin-Maldonado, Angely Ortiz-Rosado, Valerie Rodríguez-Irizarry.

**Methodology:** Marinel Ocasio-Rivera, Frances Marin-Maldonado, Geraline Trossi-Torres, Angely Ortiz-Rosado, Valerie Rodríguez-Irizarry, Eric Rodriguez-Lopez, Edu B Suarez-Martínez.

**Project administration:** Frances Marin-Maldonado, Geraline Trossi-Torres, Angely Ortiz-Rosado, Eric Rodriguez-Lopez, Edu B Suarez-Martínez.

**Resources:** Edu B Suarez-Martínez.

**Software:** Marinel Ocasio-Rivera, Frances Marin-Maldonado, Geraline Trossi-Torres, Angely Ortiz-Rosado, Valerie Rodríguez-Irizarry, Eric Rodriguez-Lopez.

**Supervision:** Frances Marin-Maldonado, Geraline Trossi-Torres, Edu B Suarez-Martínez.

**Validation:** Edu B Suarez-Martínez.

**Visualization:** Edu B Suarez-Martínez.

**Writing – original draft:** Marinel Ocasio-Rivera.

**Writing – review & editing:** Sharilyn Almodovar, Edu B Suarez-Martínez.
